# Non-neoplastic intracranial cystic lesions: not everything is an arachnoid cyst

**DOI:** 10.1590/0100-3984.2019.0144

**Published:** 2021

**Authors:** Ronaldo Gonçalves Pereira, Bruno Niemeyer de Freitas Ribeiro, Rafael Teixeira de Lima Hollanda, Letícia Baldez de Almeida, Thalita Baptista Simeão, Edson Marchiori

**Affiliations:** 1 Hospital Casa de Portugal / 3D Diagnóstico por Imagem, Rio de Janeiro, RJ, Brazil.; 2 Instituto Estadual do Cérebro Paulo Niemeyer, Rio de Janeiro, RJ, Brazil.; 3 Universidade Federal do Rio de Janeiro (UFRJ), Rio de Janeiro, RJ, Brazil.

**Keywords:** Arachnoid cysts/diagnosis, Brain/diagnostic imaging, Cysts/diagnostic imaging, Epidermal cyst/diagnostic imaging, Magnetic resonance imaging, Cistos aracnoides/diagnóstico, Encéfalo/diagnóstico por imagem, Cistos/diagnóstico por imagem, Cisto epidérmico/diagnóstico por imagem, Ressonância magnética

## Abstract

Intracranial cystic lesions are common findings on neuroimaging examinations, arachnoid cysts being the most common type of such lesions. However, various lesions of congenital, infectious, or vascular origin can present with cysts. In this pictorial essay, we illustrate the main causes of non-neoplastic intracranial cystic lesions, discussing their possible differential diagnoses as well as their most relevant imaging aspects.

## Abstract (pt)

Lesões císticas intracranianas são achados comuns nos exames de neuroimagem, sendo os cistos aracnoides os mais comuns. Porém, muitas outras lesões, de origem congênita, infecciosa e vascular, podem se apresentar com cistos. Neste ensaio ilustramos as principais causas de lesões císticas intracranianas não neoplásicas, discutindo seus possíveis diagnósticos diferenciais, bem como seus aspectos de imagem mais relevantes.

## INTRODUCTION

Intracranial cysts, which are typically asymptomatic, are common findings on neuroimaging examinations. Arachnoid cysts are the most common type, accounting for 1% of all intracranial masses, characterized by well-defined extra-axial lesions, with aspects similar to those of the cerebrospinal fluid (CSF) in all magnetic resonance imaging (MRI) sequences, and rarely provoke symptoms. There is usually a reduction in the brain parenchyma adjacent to the region of the cyst. The most common location for an arachnoid cyst in the temporal region. It has also been suggested that such cysts are associated with temporal lobe hypogenesis^(1)^. However, countless other lesions, including those of congenital, infectious, or vascular origin, can be associated with intra-axial and extra-axial cystic changes.

A series of recent studies conducted in Brazil have highlighted the importance of MRI in the assessment of diseases of the nervous system^(2-7)^. In this pictorial essay, we illustrate the main causes of non-neoplastic intracranial cystic lesions, discussing their possible differential diagnoses as well as their most relevant imaging aspects.

## CONGENITAL INTRACRANIAL CYSTS

### Epidermoid cysts

**Figure 1:**
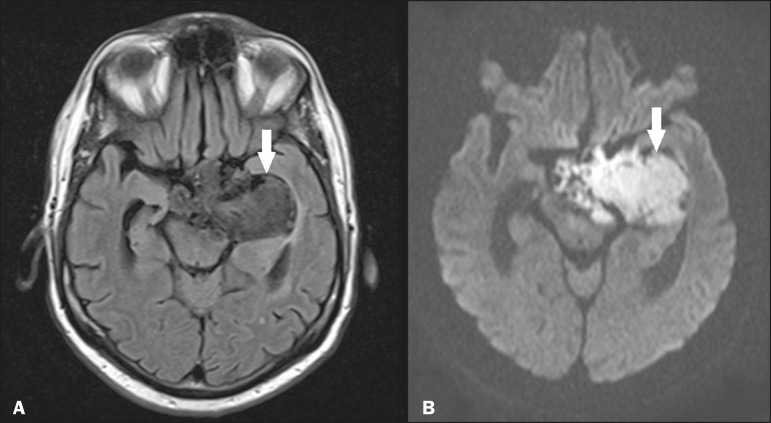
Epidermoid cyst. MRI showing a cyst-like formation in the suprasellar and left juxtasellar region, showing a hypointense signal in a FLAIR sequence (A - arrow) and a hyperintense signal in a functional diffusion-weighted sequence (B - arrow).

Epidermoid cysts represent a congenital disorder of ectodermal origin and are slow-growing lesions. The most common locations are the cerebellopontine angle cistern, the sellar/suprasellar region, the temporal fossa, and the quadrigeminal cistern. On MRI, an epidermoid cyst shows a signal intensity similar to that of the CSF, albeit with restricted diffusion (Figure 1). The main differential diagnosis is with an arachnoid cyst, and the distinction is made by evaluating images acquired in fluid-attenuated inversion recovery (FLAIR) sequences, in which an epidermoid cyst shows heterogeneous areas of high signal intensity in relation to the CSF (a so-called “dirty” signal).

### Pineal cyst

**Figure 2:**
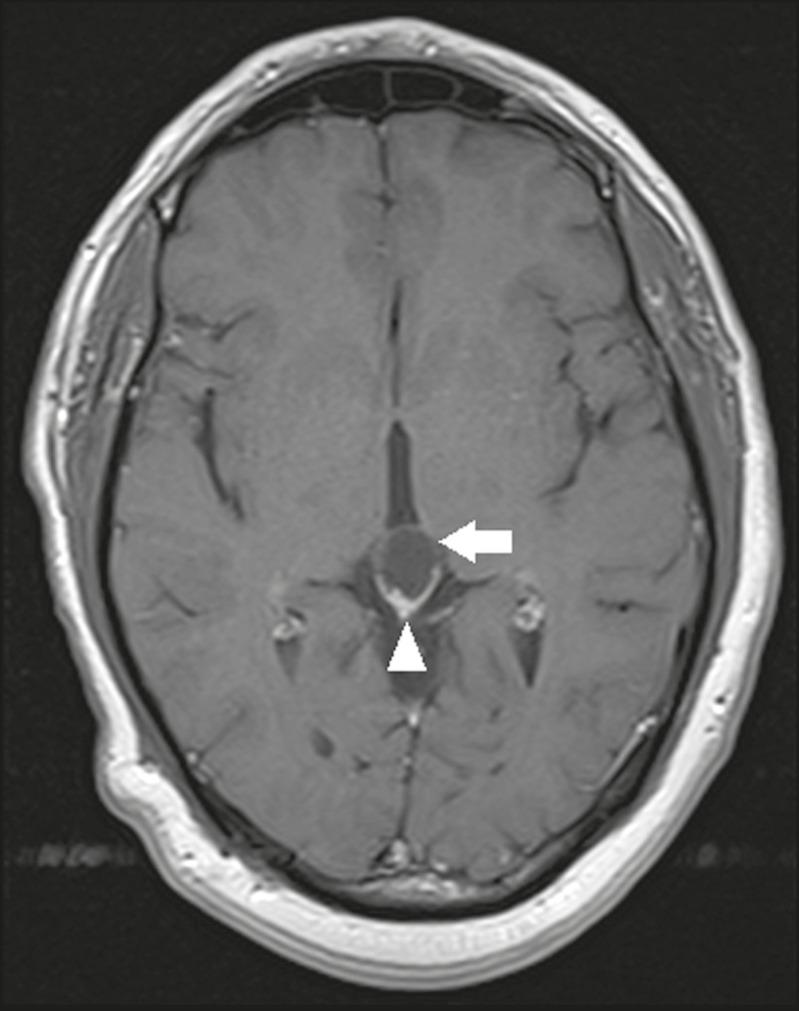
Pineal cyst. Contrast-enhanced axial T1-weighted MRI showing a cyst-like formation in the pineal gland region, with no evidence of septations or mural nodules (arrow), with a thin, smooth line of contrast enhancement along the margin (arrowhead).

A pineal cyst is defined as a unilocular cyst within the pineal gland, with a signal similar to that of the CSF. Most pineal cysts (80%) are smaller than 1 cm in diameter; those larger than 1.5 cm can result in hydrocephalus due to compression of the aqueduct of Sylvius. In most cases, pineal cysts present peripheral contrast enhancement, due to the pineal parenchyma, and some contain calcifications. There can be extravasation of the contrast agent, the enhancement simulating a solid lesion, because the pineal parenchyma of the cyst lining has no blood-brain barrier (Figure 2).

### Glial cyst

**Figure 3:**
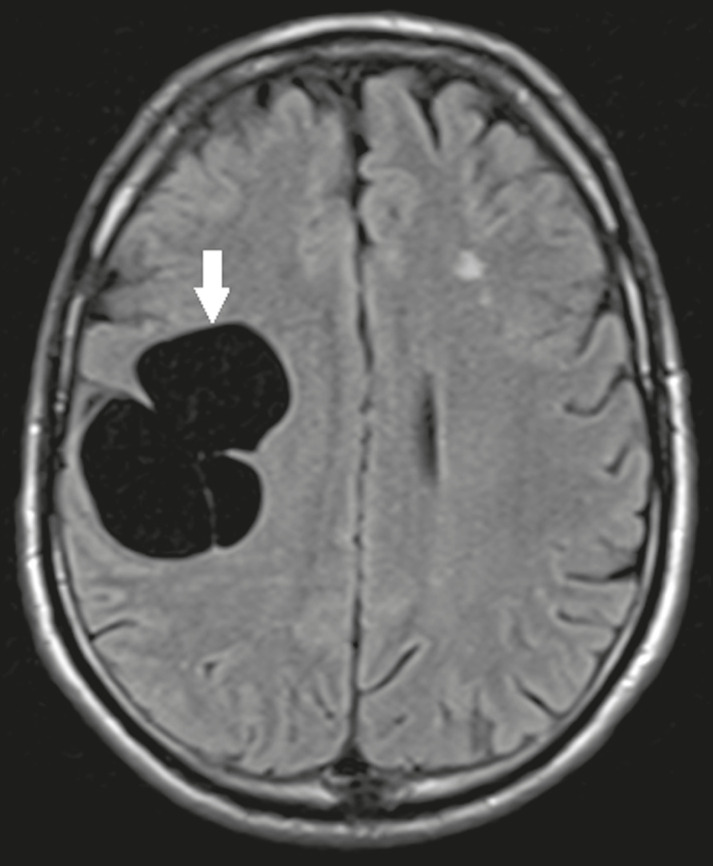
Neuroglial cyst. Axial FLAIR MRI sequence showing a cyst-like formation in the white matter of the right frontal lobe with signal intensity similar to that of the CSF and without surrounding edema (arrow).

A glial cyst is a rare congenital lesion, covered with glial cells and located within the white matter. Glial cysts are typically unilocular and well-defined, with smooth, rounded margins. On MRI, the signal intensity of a glial cyst is comparable to that of the CSF and there is no surrounding edema. Glial cysts can affect any part of the neuraxis. They can be intra-axial or extra-axial, the frontal lobe being the area most often affected^(8)^, as illustrated in Figure 3. One of the main differential diagnoses is a porencephalic cyst, which communicates with the lateral ventricle or subarachnoid space and usually presents with surrounding gliosis.

### Rathke’s cleft cysts

**Figure 4:**
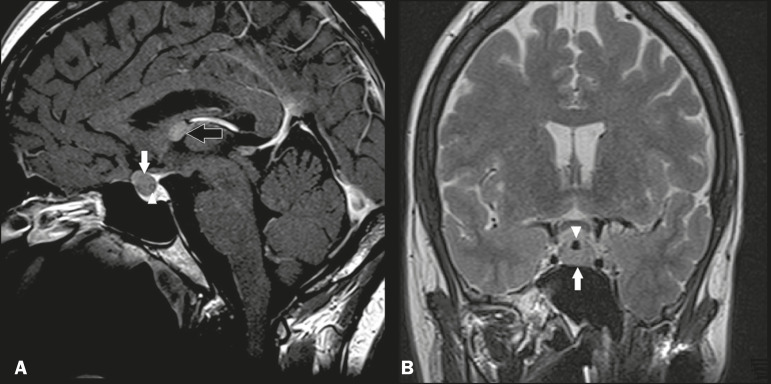
Rathke’s cleft cyst. Contrast-enhanced sagittal T1-weighted MRI (**A**) and coronal T2-weighted MRI (**B**), showing an expansile cystic lesion extending slightly into the suprasellar region, without contrast enhancement (white arrow). Note the small intracystic nodule (arrowhead), as well as the colloid cyst in the third ventricle (black arrow).

Cysts arising from the embryological remnants of Rathke’s pouch are generally asymptomatic, and the incidence of such cysts is higher in females. In most cases, Rathke’s cleft cysts are located in the pars intermedia, although the suprasellar region can also be affected. On MRI, the intensity of the signal is variable, depending on the content of the cyst, with no contrast uptake, and in some cases there can be a small intracystic nodule^(9)^, as depicted in Figure 4. A solid component with a hyperintense signal on T1-weighted images and a hypointense signal on T2-weighted images, seen in 45% of cases, is the aspect most suggestive of the diagnosis. Calcification is uncommon.

### Colloid cysts

**Figure 5:**
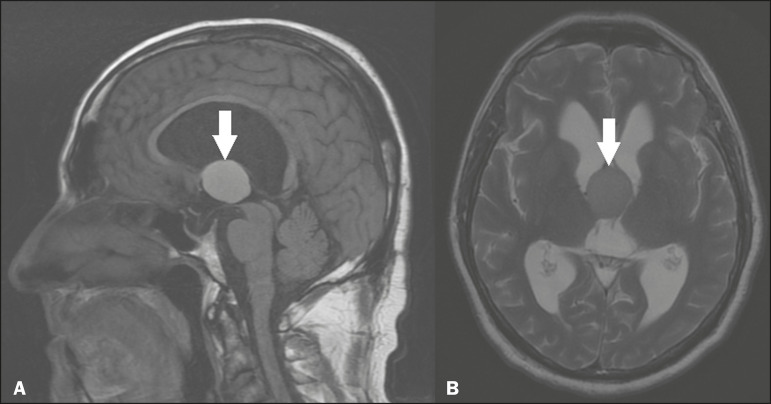
Colloid cyst. Sagittal T1-weighted MRI (**A**) and axial T2-weighted MRI (**B**), showing a cyst-like formation in the anterosuperior portion of the third ventricle, with a signal that was hyperintense in the T1-weighted sequence and hypointense in the T2-weighted sequence (arrows). Note the hydrocephalus.

Colloid cysts are classically located in the anterosuperior portion of the third ventricle, adjacent to the foramen of Monro. When rich in proteins and cholesterol, they show an MRI signal that is hyperintense on T1-weighted images and hypointense on T2-weighted images, rarely showing contrast enhancement, which is typically peripheral when it occurs. Acute obstructive hydrocephalus, albeit rare, has been reported and can occur even in small cysts^(9,10)^, as shown in Figure 5.

### Dermoid cysts

**Figure 6:**
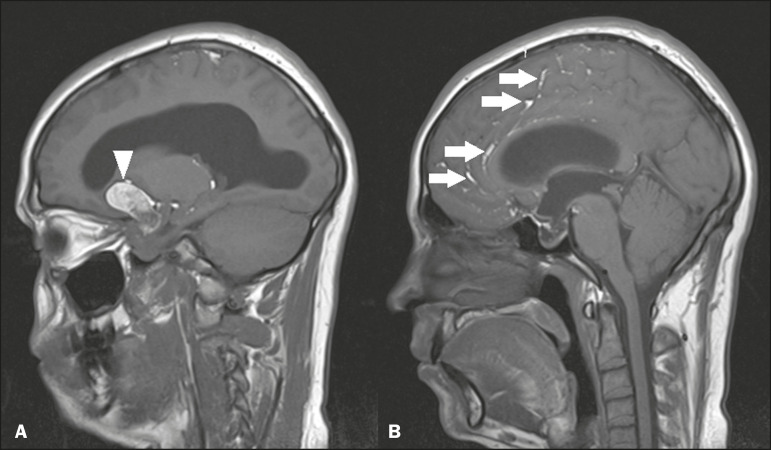
Dermoid cyst. Sagittal T1-weighted MRI sequences (**A** and **B**), showing a heterogeneous formation, with a signal that was predominantly hyperintense (arrowhead), located in the juxtasellar region. In B, hyperintense foci were also seen scattered throughout the cortical sulci (arrows), characteristic of a ruptured dermoid cyst, together with injury to the suprasellar cistern.

Dermoid cysts are uncommon and present as lobulated, well-defined, heterogeneous masses in the midline, showing an MRI signal that is hyperintense on T1-weighted images and variable on T2-weighted images, secondary to the presence of fat. They do not typically show contrast enhancement. In rare cases, they can exert a mass effect, with compression of the optical chiasm, or can rupture. In ruptured cysts, the most common finding is that of foci with MRI signals that are hyperintense on T1-weighted images, throughout the subarachnoid space and sometimes also in the ventricles. Leptomeningeal enhancement is indicative of aseptic meningitis^(9)^, as illustrated in Figure 6.

## CYSTIC LESIONS SECONDARY TO INFECTION

### Cerebral cryptococcosis

**Figure 7:**
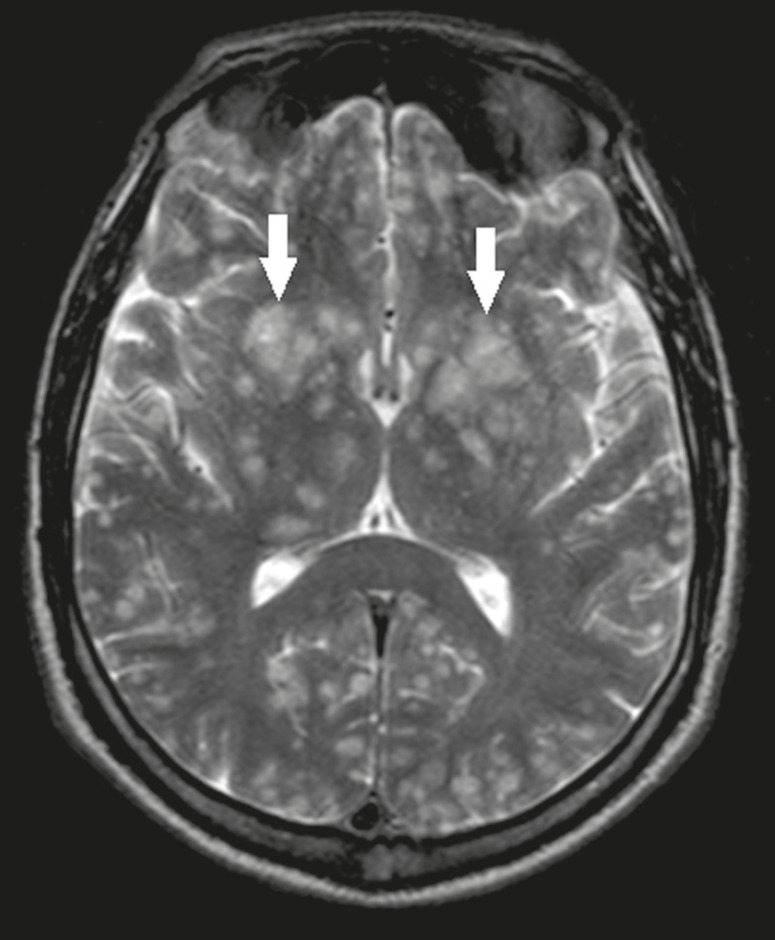
Cerebral cryptococcosis. Axial T2-weighted MRI showing hyperintense, cyst-like formations with a soap-bubble appearance, distributed throughout the basal ganglia, in the periventricular and subcortical white matter (arrows), featuring gelatinous pseudocysts.

Cerebral cryptococcosis is a fungal infection caused by inhalation of *Cryptococcus neoformans* spores and typically affects immunocompromised patients. Infection with *C. neoformans* var. *neoformans* is seen in immunosuppressed individuals, whereas infection with *C. neoformans* var. *gattii* can be seen in individuals without known immunodeficiency. The types of involvement include meningitis, cryptococcoma, gelatinous pseudocysts (with a soap-bubble appearance), plexitis, ventriculitis, empyema, miliary nodules. Pseudocysts are secondary to the filling of the perivascular space with the glycosaminoglycans produced by the fungus and are commonly characterized, on MRI, by multiple lesions dispersed throughout the cerebral parenchyma, most often in the basal ganglia (near the anterior commissure), with a hyperintense signal on T2-weighted images; restricted diffusion may be present in a few cases, and there is no or minimal contrast enhancement^(11-13)^, as shown in Figure 7.

### Neurocysticercosis

**Figure 8:**
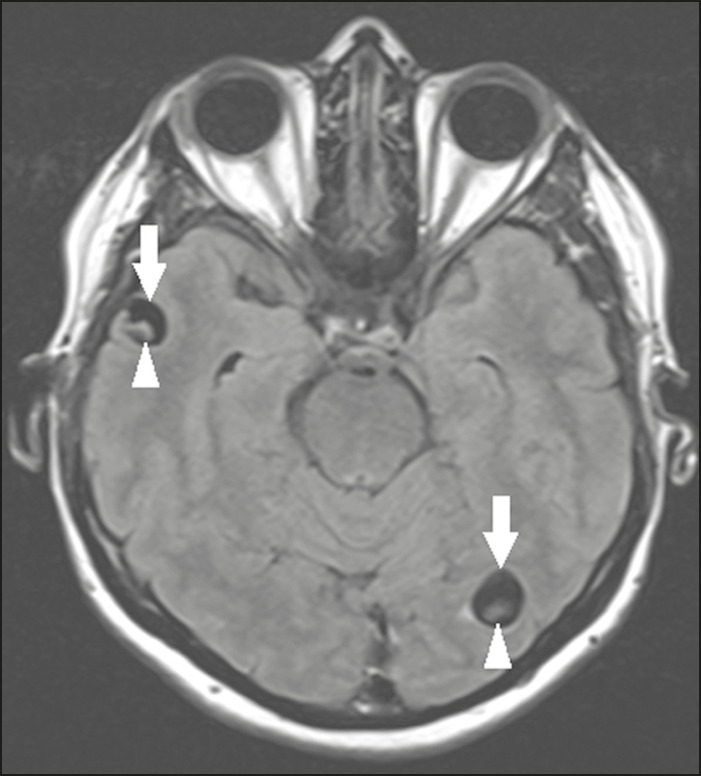
Neurocysticercosis. Axial FLAIR MRI showing cyst-like formations with signal intensities similar to that of the CSF, affecting the right temporal and left occipital lobes (arrows), and containing scolices (arrowheads). There is no evidence of perilesional edema, the absence of which is characteristic of the vesicular stage.

The most common parasitic infection of the central nervous system, neurocysticercosis is caused by the larva (cysticercus) of the tapeworm *Taenia solium*. The cysticerci can present in a cystic or racemic form. The cystic form is divided into four stages, the vesicular stage being characterized by a cyst containing a scolex, without enhancement and without perilesional edema (Figure 8), and the colloidal vesicular stage being characterized by enhancement of the cystic walls and perilesional edema. The racemic form is characterized by lesions in clusters, with numerous grouped vesicles, which can obliterate the basal cisterns, sylvian fissure, and interhemispheric fissure. On MRI, it presents as a multicystic lesion, with a signal similar to that of the CSF on T1- and T2-weighted images, without restricted diffusion and without significant enhancement by gadolinium, and it is unusual for an individual scolex to be visualized. The use of strongly T2-weighted sequences, such as constructive interference in steady state sequences and fast imaging employing steady-state acquisition sequences, is considered the ideal method for visualizing the scolex^(11)^.

### Echinococcosis

**Figure 9:**
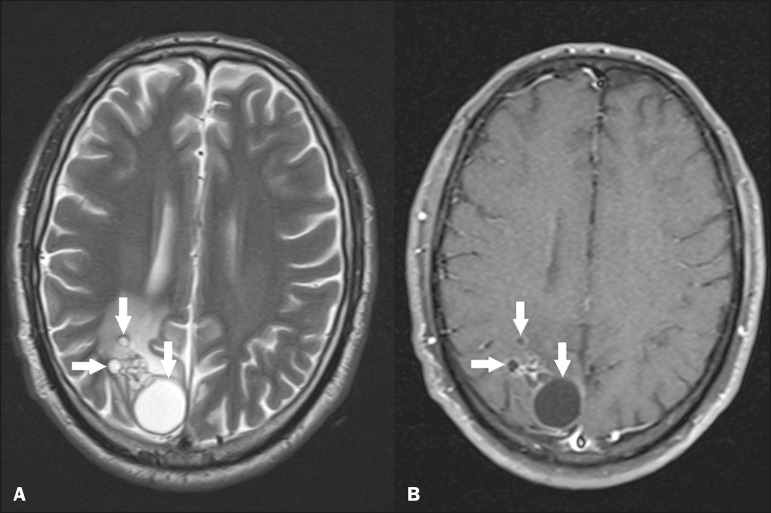
Alveolar echinococcosis. Axial T2-weighted MRI (**A**) and contrast-enhanced axial T1-weighted MRI (**B**), showing multiple coalescent cyst-like lesions in the right occipital lobe, characterized by a signal similar to that of the CSF in the T2-weighted sequence and peripheral contrast enhancement (arrows).

Echinococcosis is caused by infection with *Echinococcus granulosus* or *Echinococcus multilocularis*. Infection with *E. granulosus* is known as hydatidosis and presents as a single cyst, isointense to the CSF in T1- and T2-weighted MRI sequences. Infection with *E. multilocularis* is known as alveolar echinococcosis and presents as multiple irregular cysts, which usually show contrast enhancement^(14)^, as depicted in Figure 9.

### Congenital cytomegalovirus infection

Caused by infection with viruses of the herpesvirus family, congenital cytomegalovirus is the most common congenital infection. It is more likely when maternal infection occurs in the first or second trimester of pregnancy, especially when it occurs in the first trimester. The imaging findings of congenital cytomegalovirus infection include microcephaly with ventriculomegaly, volume loss of white matter, delayed myelination, calcifications typically located in the periventricular region, and cysts, especially in the anterior temporal lobe^(15)^. A finding of cysts in the temporal lobe indicates a differential diagnosis that is quite common in the field of neuroradiology. Imaging findings that can confuse the diagnosis include enlarged perivascular spaces, ischemic lesions, neuroepithelial cysts, and focal deformations in the temporal horn of the lateral ventricle. Expansile primary and metastatic tumors can be difficult to identify because of the anatomical characteristics of the region. These are some of the factors that call for a specific study of the differential diagnosis of cystic lesions and normal structures of cystic aspect that can be found in the temporal lobes.

## VASCULAR CYSTS AND CYST-LIKE LESIONS

### Porencephalic cysts

**Figure 10:**
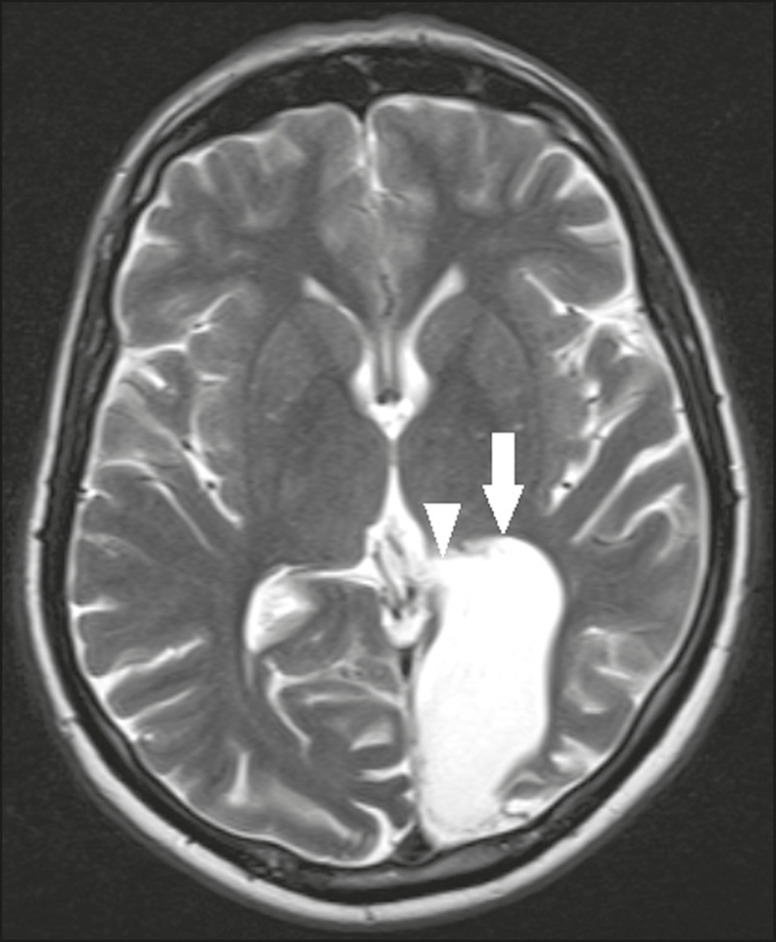
Porencephalic cyst. Axial T2-weighted MRI showing a cyst-like lesion in the left occipital lobe, with a signal intensity similar to that of the CSF (arrow). Note the communication with the ventricular system (arrowhead) and that the edges of the lesion are composed of white matter.

A porencephalic cyst is a congenital disorder of vascular origin resulting from intrauterine ischemia. It presents as an intraparenchymal area of cystic degeneration, filled with CSF and covered with a white substance, which can include communication with the ventricular system or subarachnoid space (Figure 10).

### Neonatal hypoxic-ischemic encephalopathy

**Figure 11:**
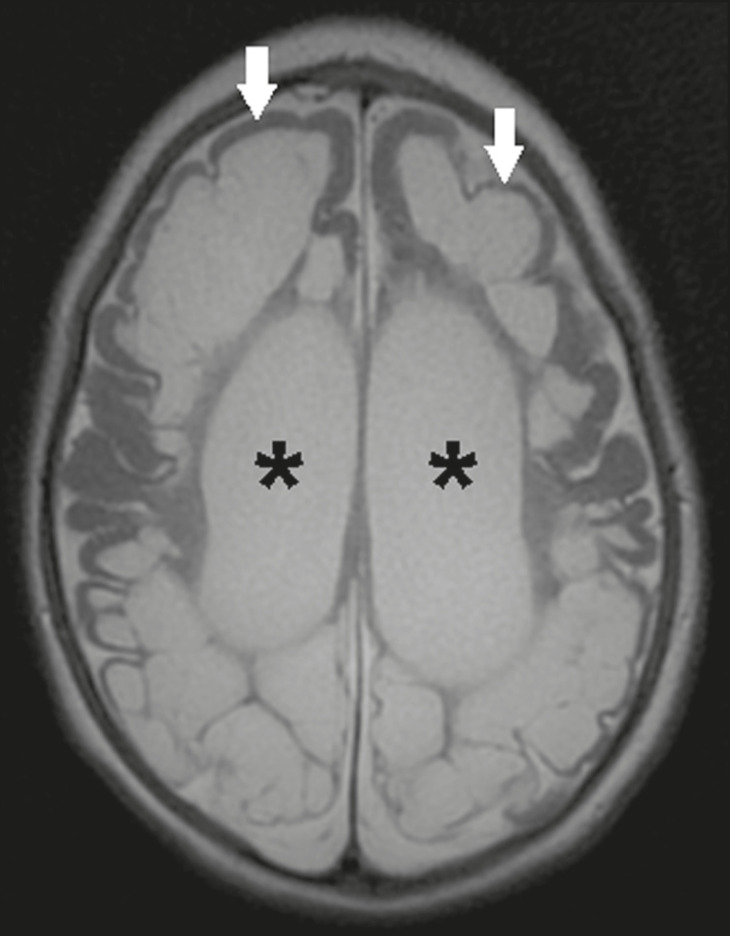
Neonatal hypoxic-ischemic encephalopathy. Axial T2-weighted MRI showing multiple cysts of varying sizes, with intensity similar to that of the CSF, distributed bilaterally in the white matter (arrows). Note the dilatation of the lateral ventricles (asterisks), secondary to involvement of the cerebral parenchyma.

Neonatal ischemic hypoxemia can manifest in different ways, depending on the severity, time of hypoxia, and age of the infant. The typical patterns, in terms of the timing of the insult, are as follows: hydranencephaly and porencephaly, in those born at ≤ 28 weeks of gestation; periventricular leukomalacia, together with lesions of the basal ganglia or thalamic nuclei, in those born at 32-36 weeks; and multicystic encephalomalacia, in those born at 39-40 weeks. Multicystic encephalomalacia presents as multiple, bilateral, symmetrical cysts, of varying sizes, affecting the white matter while sparing the infratentorial region, cerebellum, and medulla^(16)^, as illustrated in Figure 11.

### Enlarged perivascular spaces

**Figure 12:**
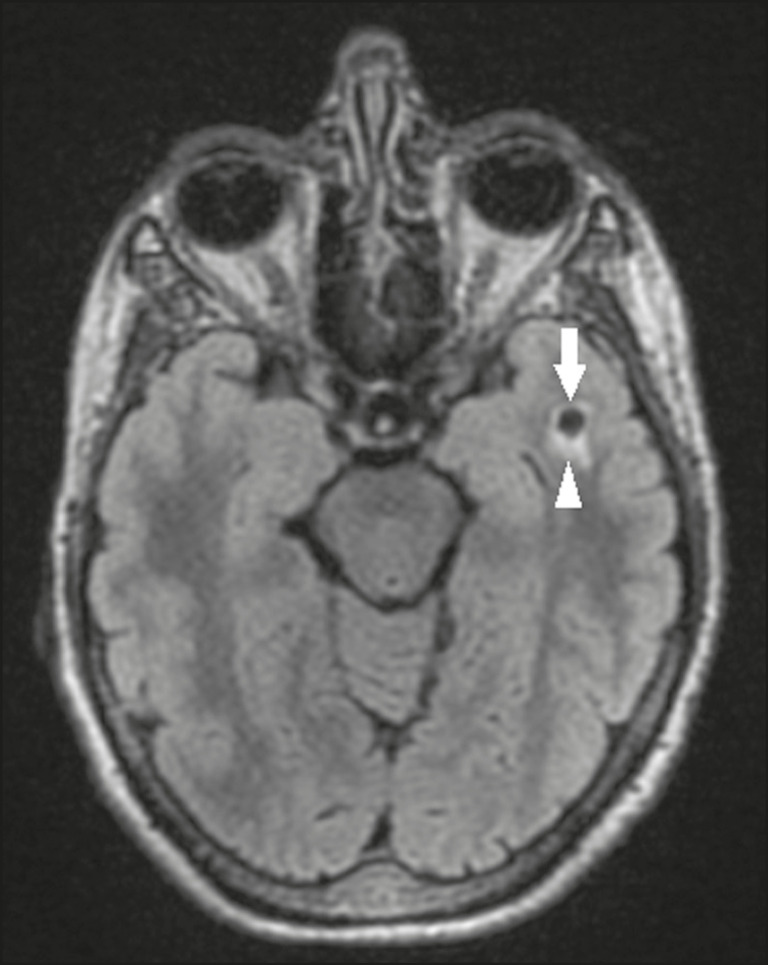
Enlarged perivascular space. Axial FLAIR MRI showing a small cyst-like lesion in the left temporal lobe with a signal similar to that of the CSF (arrow). Note the slight hyperintensity in the surrounding brain parenchyma (arrowhead).

Perivascular spaces are structures covered by the pia mater that accompany the vessels on their way from the subarachnoid space to the interior of the brain parenchyma. When swollen, they are referred to as enlarged perivascular spaces. The perivascular spaces of the temporal lobe are variants of the enlarged perivascular spaces, which can mimic cystic tumors such as dysembryoplastic neuroepithelial tumors (Figure 12).

## OTHER CYSTS AND CYST-LIKE LESIONS

### Arachnoid granulations

**Figure 13:**
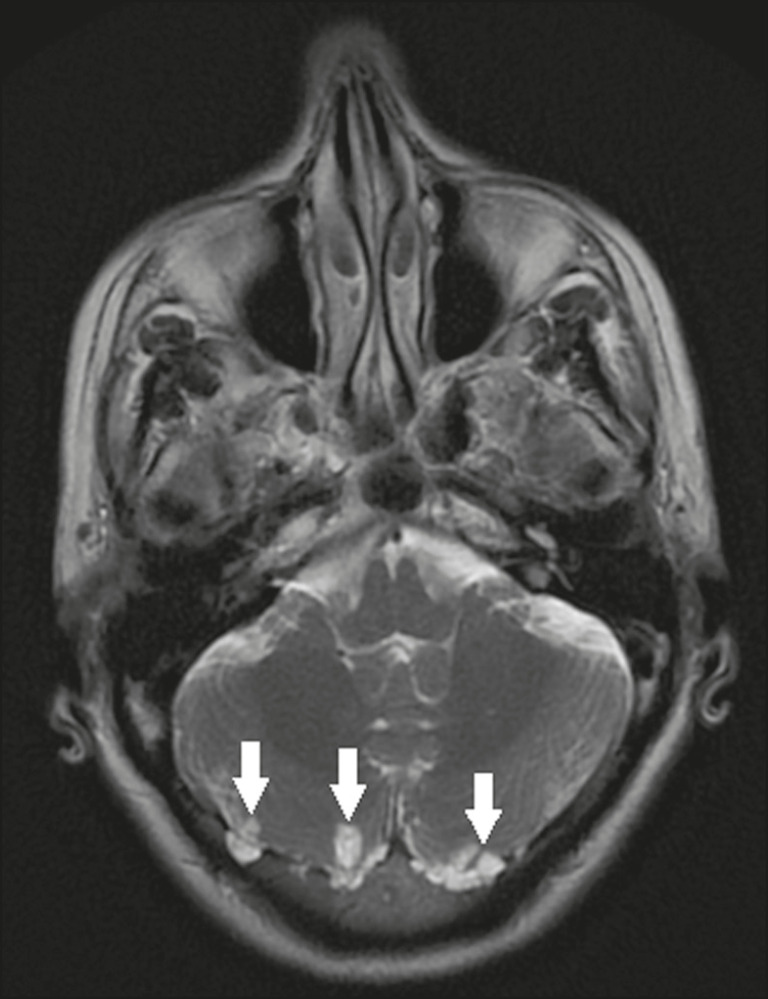
Arachnoid granulations. Axial T2-weighted MRI showing rounded, cyst-like lesions, located extra-axially, with intensity similar to that of the CSF, in close proximity to the cerebellar hemispheres (arrows).

Arachnoid granulations are projections of the arachnoid membrane into the dural sinuses and allow the CSF to enter the subarachnoid space. They often have a parasagittal location, at the confluence of the sinuses. The absence of contrast filling can erroneously be interpreted as a possible cerebral venous thrombosis^(17)^ (Figure 13).

### Hereditary metabolic disorders

**Figure 14:**
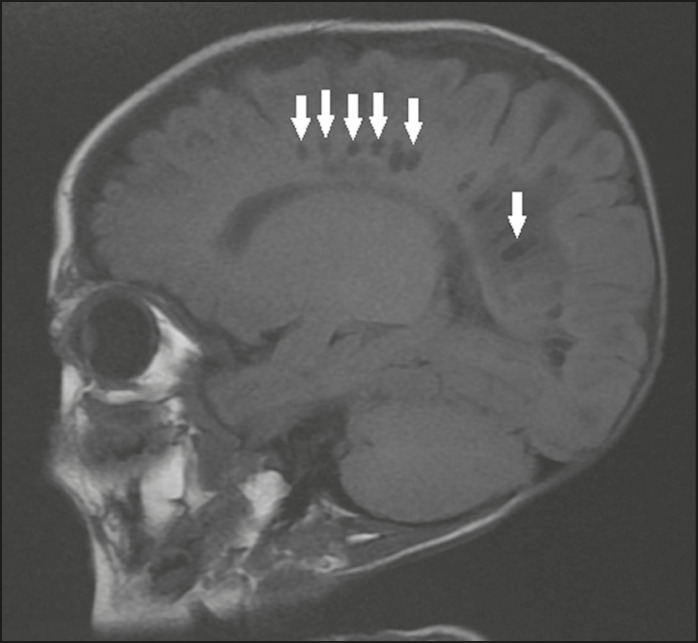
Mucopolysaccharidosis. T1-weighted sagittal MRI showing multiple cystic images with a signal similar to CSF, affecting the region around the corpus callosum (arrows), resulting in enlarged perivascular spaces.

Various hereditary metabolic disorders, including mucopolysaccharidosis, Labrune syndrome, cystic leukoencephalopathy without megalencephaly, and mitochondrial encephalopathy, can result in the development of cysts (Figure 14). In general, such disorders arise in the first or second decades of life, affecting the white matter in different ways, depending on the disorder in question and the age at onset. Other findings that may be present include the following^(18)^: spinal cord compression resulting from changes in the craniocervical junction, in mucopolysaccharidosis; parietal enhancement of the cysts and parenchymal calcifications, in Labrune syndrome; and acute or chronic infarctions that can be accompanied by a lactate peak on proton spectroscopy, in mitochondrial encephalopathies.

## CONCLUSION

A wide spectrum of diseases can cause intracranial cystic lesions, making them a diagnostic challenge. To make the differential diagnosis, it is of paramount importance to consider imaging findings in conjunction with the clinical history.
